# Effects of BMI, Fat Mass, and Lean Mass on Asthma in Childhood: A Mendelian Randomization Study

**DOI:** 10.1371/journal.pmed.1001669

**Published:** 2014-07-01

**Authors:** Raquel Granell, A. John Henderson, David M. Evans, George Davey Smith, Andrew R. Ness, Sarah Lewis, Tom M. Palmer, Jonathan A. C. Sterne

**Affiliations:** 1 School of Social and Community Medicine, University of Bristol, Bristol, United Kingdom; 2 MRC Integrative Epidemiology Unit, University of Bristol, Bristol, United Kingdom; 3 University of Queensland Diamantina Institute, Translational Research Institute, Brisbane, Australia; 4 UK National Institute for Health Research Bristol Nutrition Biomedical Research Unit in Nutrition, Diet and Lifestyle, University Hospitals Bristol NHS Foundation Trust and University of Bristol, Bristol, United Kingdom; 5 School of Oral and Dental Sciences, University of Bristol, Bristol, United Kingdom; 6 Division of Health Sciences, Warwick Medical School, University of Warwick, Coventry, United Kingdom; Imperial College London, United Kingdom

## Abstract

**Background:**

Observational studies have reported associations between body mass index (BMI) and asthma, but confounding and reverse causality remain plausible explanations. We aim to investigate evidence for a causal effect of BMI on asthma using a Mendelian randomization approach.

**Methods and Findings:**

We used Mendelian randomization to investigate causal effects of BMI, fat mass, and lean mass on current asthma at age 7½ y in the Avon Longitudinal Study of Parents and Children (ALSPAC). A weighted allele score based on 32 independent BMI-related single nucleotide polymorphisms (SNPs) was derived from external data, and associations with BMI, fat mass, lean mass, and asthma were estimated. We derived instrumental variable (IV) estimates of causal risk ratios (RRs). 4,835 children had available data on BMI-associated SNPs, asthma, and BMI. The weighted allele score was strongly associated with BMI, fat mass, and lean mass (all *p*-values<0.001) and with childhood asthma (RR 2.56, 95% CI 1.38–4.76 per unit score, *p* = 0.003). The estimated causal RR for the effect of BMI on asthma was 1.55 (95% CI 1.16–2.07) per kg/m^2^, *p* = 0.003. This effect appeared stronger for non-atopic (1.90, 95% CI 1.19–3.03) than for atopic asthma (1.37, 95% CI 0.89–2.11) though there was little evidence of heterogeneity (*p* = 0.31). The estimated causal RRs for the effects of fat mass and lean mass on asthma were 1.41 (95% CI 1.11–1.79) per 0.5 kg and 2.25 (95% CI 1.23–4.11) per kg, respectively. The possibility of genetic pleiotropy could not be discounted completely; however, additional IV analyses using *FTO* variant rs1558902 and the other BMI-related SNPs separately provided similar causal effects with wider confidence intervals. Loss of follow-up was unlikely to bias the estimated effects.

**Conclusions:**

Higher BMI increases the risk of asthma in mid-childhood. Higher BMI may have contributed to the increase in asthma risk toward the end of the 20th century.

*Please see later in the article for the Editors' Summary*

## Abstract 2

In this study, Granell and colleagues used Mendelian randomization to investigate causal effects of BMI, fat mass, and lean mass on current asthma at age 7½ years in the Avon Longitudinal Study of Parents and Children (ALSPAC) and found that higher BMI increases the risk of asthma in mid-childhood.

*Please see later in the article for the Editors' Summary*

## Editors' Summary

**Background:**

The global burden of asthma, a chronic (long-term) condition caused by inflammation of the airways (the tubes that carry air in and out of the lungs), has been rising steadily over the past few decades. It is estimated that, nowadays, 200–300 million adults and children worldwide are affected by asthma. Although asthma can develop at any age, it is often diagnosed in childhood—asthma is the most common chronic disease in children. In people with asthma, the airways can react very strongly to allergens such as animal fur or to irritants such as cigarette smoke, becoming narrower so that less air can enter the lungs. Exercise, cold air, and infections can also trigger asthma attacks, which can be fatal. The symptoms of asthma include wheezing, coughing, chest tightness, and shortness of breath. Asthma cannot be cured, but drugs can relieve its symptoms and prevent acute asthma attacks.

**Why Was This Study Done?:**

We cannot halt the ongoing rise in global asthma rates without understanding the causes of asthma. Some experts think obesity may be one cause of asthma. Obesity, like asthma, is increasingly common, and observational studies (investigations that ask whether individuals exposed to a suspected risk factor for a condition develop that condition more often than unexposed individuals) in children have reported that body mass index (BMI, an indicator of body fat calculated by dividing a person's weight in kilograms by their height in meters squared) is positively associated with asthma. Observational studies cannot prove that obesity causes asthma because of “confounding.” Overweight children with asthma may share another unknown characteristic (confounder) that actually causes both obesity and asthma. Moreover, children with asthma may be less active than unaffected children, so they become overweight (reverse causality). Here, the researchers use “Mendelian randomization” to assess whether BMI has a causal effect on asthma. In Mendelian randomization, causality is inferred from associations between genetic variants that mimic the effect of a modifiable risk factor and the outcome of interest. Because gene variants are inherited randomly, they are not prone to confounding and are free from reverse causation. So, if a higher BMI leads to asthma, genetic variants associated with increased BMI should be associated with an increased risk of asthma.

**What Did the Researchers Do and Find?:**

The researchers investigated causal effects of BMI, fat mass, and lean mass on current asthma at age 7½ years in 4,835 children enrolled in the Avon Longitudinal Study of Parents and Children (ALSPAC, a long-term health project that started in 1991). They calculated an allele score for each child based on 32 BMI-related genetic variants, and estimated associations between this score and BMI, fat mass and lean mass (both measured using a special type of X-ray scanner; in children BMI is not a good indicator of “fatness”), and asthma. They report that the allele score was strongly associated with BMI, fat mass, and lean mass, and with childhood asthma. The estimated causal relative risk (risk ratio) for the effect of BMI on asthma was 1.55 per kg/m^2^. That is, the relative risk of asthma increased by 55% for every extra unit of BMI. The estimated causal relative risks for the effects of fat mass and lean mass on asthma were 1.41 per 0.5 kg and 2.25 per kg, respectively.

**What Do These Findings Mean?:**

These findings suggest that a higher BMI increases the risk of asthma in mid-childhood and that global increases in BMI toward the end of the 20th century may have contributed to the global increase in asthma that occurred at the same time. It is possible that the observed association between BMI and asthma reported in this study is underpinned by “genetic pleiotropy” (a potential limitation of all Mendelian randomization analyses). That is, some of the genetic variants included in the BMI allele score could conceivably also increase the risk of asthma. Nevertheless, these findings suggest that public health interventions designed to reduce obesity may also help to limit the global rise in asthma.

**Additional Information:**

Please access these websites via the online version of this summary at http://dx.doi.org/10.1371/journal.pmed.1001669.

- The US Centers for Disease Control and Prevention provides information on asthma and on all aspects of overweight and obesity (in English and Spanish)
- The World Health Organization provides information on asthma and on obesity (in several languages)
- The UK National Health Service Choices website provides information about asthma, about asthma in children, and about obesity (including real stories)
- The Global Asthma Report 2011 is available
- The Global Initiative for Asthma released its updated Global Strategy for Asthma Management and Prevention on World Asthma Day 2014
- Information about the Avon Longitudinal Study of Parents and Children is available
- MedlinePlus provides links to further information on obesity in children, on asthma, and on asthma in children (in English and Spanish
- Wikipedia has a page on Mendelian randomization (note: Wikipedia is a free online encyclopedia that anyone can edit; available in several languages)

## Introduction

The prevalence of both obesity and asthma has increased in children [1], leading to speculation that adiposity and asthma may be causally related. Observational studies in children have reported that obesity is positively associated with asthma [2],[3], and longitudinal studies have showed that obesity precedes incident asthma [4],[5]. Most such studies were based on body mass index (BMI; weight [kg] divided by height squared [m^2^]), a readily obtainable measure of body size that is essentially independent of height. However, BMI is a relatively insensitive indicator of adiposity compared with other measures, such as percent body fat estimated by skinfold thickness [6]. BMI, which is a composite of fat and lean mass, changes substantially with age during childhood [7], and the proportion and distribution of body fat differ between males and females with the same BMI [8]. Plausible explanations for a link between adiposity and asthma include mediation through mechanical, immunological, or endocrine pathways; increased perception of symptoms; genetic pleiotropy; and co-morbidity due to chronic inflammatory mechanisms.

Whether observed associations arise from a causal effect of BMI on asthma remains unclear, and the possibility of confounding by common socio-economic, lifestyle, and dietary factors, or reverse causality (for example, via children with asthma being more likely to be sedentary), remains. The identification of genetic variants related to BMI in recently reported genome-wide association studies [9] provides an opportunity to examine the evidence for a causal link, using the “Mendelian randomization” approach [10], in which evidence for a causal link between a phenotype and a disease outcome such as asthma is inferred from the associations of genetic variants with the phenotype and with the disease outcome. This approach should be unaffected by confounding or reverse causation because genetic variants are generally unrelated to confounding factors [11] and do not change after conception.

This study had three aims: (1) to investigate the association of BMI, fat mass, and lean mass with childhood asthma in a large, population-based birth cohort, (2) to use a Mendelian randomization approach based on single nucleotide polymorphisms (SNPs) associated with adiposity to investigate evidence for a causal effect of these phenotypes on asthma, and (3) to investigate whether the magnitude of effect varies with gender or when asthma is classified as atopic and non-atopic.

## Methods

### Cohort Description

The Avon Longitudinal Study of Parents and Children (ALSPAC) is a longitudinal, population-based birth cohort study that recruited 14,541 pregnant women resident in Avon, UK, with an expected date of delivery from 1 April 1991 to 31 December 1992. There were 14,541 initial pregnancies for which the mother enrolled in the ALSPAC study and either returned at least one questionnaire or attended a “Children in Focus” clinic by 19 July 1999. For these initial pregnancies, there was a total of 14,676 fetuses, resulting in 14,062 live births and 13,988 children who were alive at 1 y of age.

When the oldest children were approximately 7 y of age, an attempt was made to bolster the initial sample with eligible cases who had failed to join the study originally. The total sample size for analyses using any data collected after the age of 7 y is therefore 15,247 pregnancies, resulting in 15,458 fetuses. Of this total sample of 15,458 fetuses, 14,775 were live births and 14,701 were alive at 1 y of age. The phases of enrollment are described in more detail in the cohort profile paper [12]. The study website (http://www.bris.ac.uk/alspac/researchers/data-access/data-dictionary/) contains a fully searchable data dictionary with details of all the available data.

Ethical approval was obtained from the ALSPAC Ethics and Law Committee and local research ethics committees, and written informed consent was obtained for all measurements.

### Body Mass Index, Fat Mass, Lean Mass, and Asthma

Birth weight and length were measured by trained study staff or obtained from hospital records, as described previously [13]. At the ages of 7 to 9 y, children were invited to attend an annual research clinic, during which anthropometric measures, including height and weight, were taken [14]. Height was measured using a Harpenden stadiometer (Holtain), and weight was measured using Tanita TBF 305 scales (Tanita). BMI was calculated as weight (kg) divided by height squared (m^2^). At age 9 y, in addition to standard anthropometry, body composition (fat mass and lean mass) was measured using a Lunar Prodigy dual X-ray emission absorptiometry (DXA) scanner (GE Medical Systems Lunar) [15]. DXA uses two X-ray beams of different energies that are attenuated to different degrees as they pass through the body (according to the quantity and nature of the tissue) to estimate fat mass, lean mass, and bone mineral content. To adjust for differences in fat mass between females and males, and to adjust for height, the measures of fat mass and lean mass included in the analyses were calculated as the residuals from a linear regression of each on gender, height, and height squared. The standard deviation of fat mass residuals was 4.35 kg, approximately double that of BMI (2.01 kg/m^2^) and lean mass (1.71 kg) residuals. We divided the fat mass residuals by two in subsequent analyses, so that regression coefficients for BMI, fat mass, and lean mass that were of similar size reflected associations of similar strength. International Obesity Task Force [16] criteria were used to classify children as normal, overweight, or obese at 7½ y.

Current asthma at ages 7½, 11, and 14 y was defined based on parental self-report of ever doctor diagnosis of asthma from questionnaires sent to mothers at child ages 91, 128, and 166 mo, respectively, together with either (1) reported asthma or symptoms of wheezing in the previous 12 mo or (2) reported treatment for asthma in the previous 12 mo. Current asthma at age 9 y was defined based on parental report of asthma or symptoms of wheezing or treatment for asthma in the previous 12 mo. Current asthma at age 13 y was defined by parental report of asthma or symptoms of wheezing in the previous 12 mo. Parental report of ever doctor diagnosis of asthma at 15 y was also available. Atopic status was determined at age 7½ y by skin prick test response to a panel of up to 12 common allergens including house dust mite, grass pollen, cat, egg, peanut, and mixed nuts. A positive response was defined as a mean weal diameter of >2 mm with an absent response to negative control solution, and atopy was defined as a positive response to one or more of house dust mite, grass pollen, and cat. Atopic and non-atopic asthma were defined, respectively, as current asthma with and without atopy at 7½ y.

### Confounders

From questionnaires sent to the mother during pregnancy we obtained details of educational attainment (categorized into two levels, with lower level defined as educated to school leaving certificate at 16 y or lower) and smoking history. Sex of the child and birth weight (categorized as low birth weight if <2.5 kg) were obtained from delivery health care records. A postnatal maternal questionnaire at 8 mo after birth was used to ascertain environmental tobacco smoke exposure.

### Genetic Data

A total of 9,912 children were genotyped using the Illumina HumanHap550Quad genome-wide SNP genotyping platform by the Wellcome Trust Sanger Institute (Cambridge, UK) and LabCorp (Burlington, North Carolina, US). Individuals were excluded from further analysis on the basis of having incorrect gender assignments, minimal or excessive heterozygosity (<0.320 or >0.345 for the Sanger data and <0.310 or >0.330 for the LabCorp data), disproportionate levels of individual missingness (>3%), or evidence of cryptic relatedness (measured as proportion of identity by descent >0.1). The remaining individuals were assessed for evidence of population stratification by multidimensional scaling analysis and compared with HapMap II (release 22) European descent (CEU), Han Chinese, Japanese, and Yoruba reference populations; all individuals with non-European ancestry were removed to avoid population stratification; however, this step removed very few individuals given the nature of the study, and as a result had very little impact on the overall findings of the study. SNPs with a minor allele frequency of <1%, a call rate of <95%, or evidence for violations of Hardy-Weinberg equilibrium (*p*<5×10^−7^) were removed. Autosomal genotypic data were subsequently imputed using Markov Chain Haplotyping software (MACH v.1.0.16) and phased haplotype data from CEU individuals (HapMap release 22, Phase II NCBI B36, dbSNP 126) based on a cleaned dataset of 8,365 individuals and 500,527 autosomal SNPs. After imputation, all SNPs with indication of poor imputation quality (*r* ^2^<0.30) were removed.

A weighted allele score [17] was constructed from 32 SNPs found to have genome-wide evidence for association with BMI and adiposity in contemporary literature [9]. 21 of the 32 known BMI variants used in our study were imputed, and the rest were genotyped. These genotypes have previously been used in ALSPAC as an instrumental variable (IV) [18]–[21]. The dosage of the effect allele at each locus was multiplied by a SNP-specific weight (linear coefficient divided by average of 32 linear coefficients), then averaged across SNPs. SNP-specific weights were based on a previously reported meta-analysis [9] that excluded ALSPAC data. A weighted *FTO* dosage (using variant rs1558902 from the *FTO* gene) and a weighted allele score derived from the 31 other variants combined were also defined and used in additional analyses.

### Statistical Analysis

Analyses of associations of BMI, fat mass, and lean mass with asthma were done using datasets restricted to children with complete data on BMI at 7 y, asthma at 7½ y, and each of the 32 selected SNPs, with multiple births and non-white individuals excluded. Observational associations were further restricted to children with data on confounders (gender, birth weight, pre- and postnatal maternal exposure to smoking, maternal education) and height at 9 y (for associations with fat mass and lean mass).

Logistic regression models were used to estimate per-allele odds ratios (160 associations) for the individual associations between each of the 32 SNPs and confounding factors (Table S1). Mean differences for the association between all potential confounding factors and BMI/fat mass/lean mass were estimated using linear regression models. The linear coefficient for the association between the confounding factors and the weighted allele score was also estimated (Table S2).

Generalized linear models with binomial family and log link were used to estimate risk ratios (RRs) for associations of BMI, fat mass, and lean mass with asthma before and after adjusting for confounders, and to estimate RRs for the association between the weighted allele score and asthma. We also estimated these associations after stratifying by atopic status and gender. Linear regression models were used to estimate associations of the weighted allele score with BMI, fat mass, and lean mass. All analyses of BMI were stratified by gender. Likelihood ratio tests were derived for interactions of estimated effects with gender, and heterogeneity *p*-values for asthma subtype were derived using Chi-squared tests.

**Figure 1:**
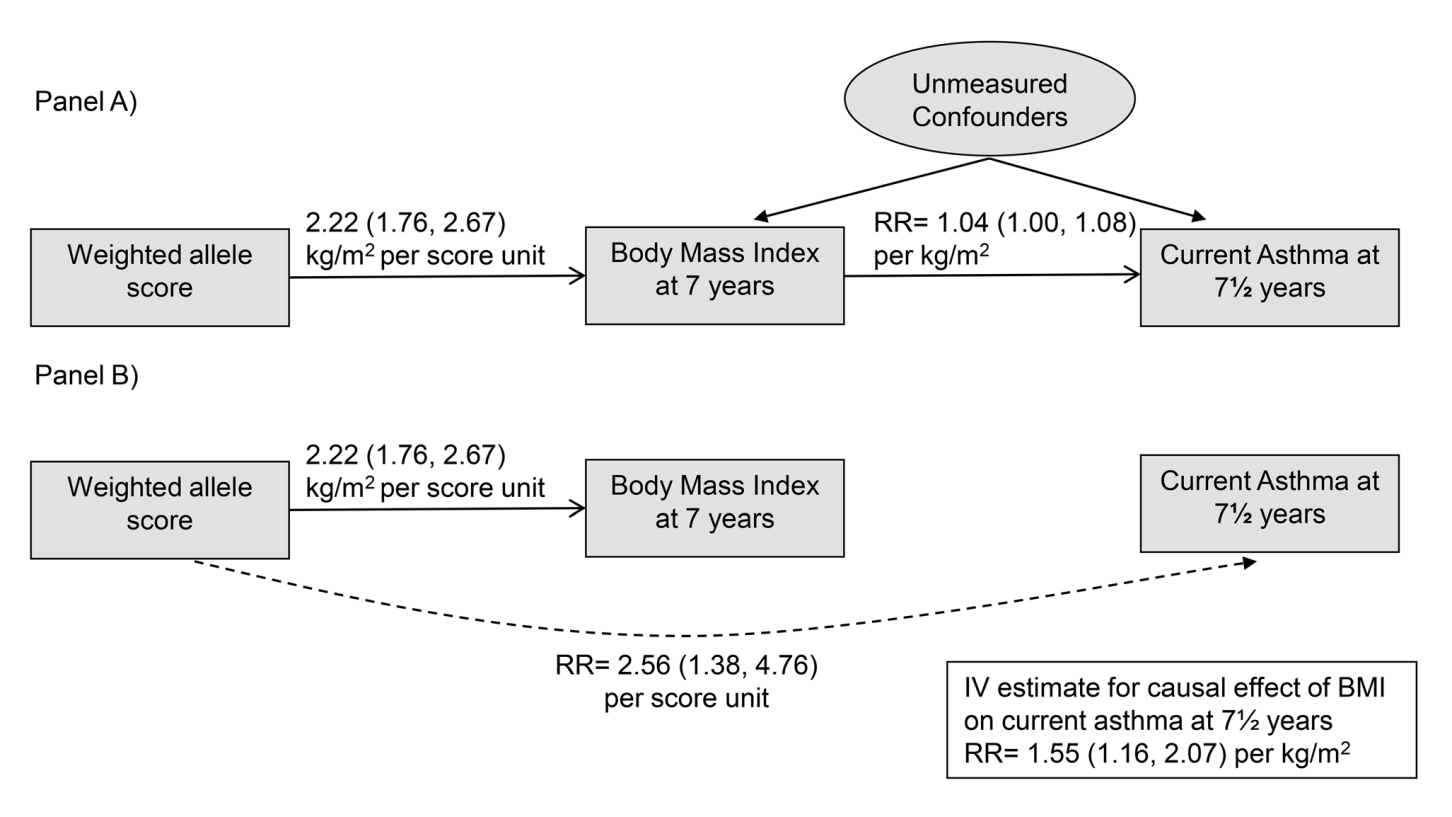
Main Mendelian randomization features and results of the study. (A) depicts the IV assumptions, and (B) shows the two unconfounded associations used to estimate causal effects of BMI on asthma: the association of the allele score with BMI and the association of the allele score with asthma.

We used two-stage IV methods [22], with genetic allele score as IV, to estimate causal RRs for effects of BMI, fat mass, and lean mass on asthma outcomes. Such methods exploit the IV assumptions (Figure 1A) that allele score is (1) associated with the phenotype (BMI), (2) independent of unmeasured confounders, and (3) independent of the outcome given the phenotype and unmeasured confounding factors. Estimation (Figure 1B) is based on the associations (1) between allele score and BMI and (2) between allele score and asthma, each of which is unconfounded based on the IV assumptions. The first stage of estimation fits a linear regression of the phenotype on the weighted allele score. The second stage is a regression of the asthma outcome on the phenotype values predicted by the first stage regression. To adjust standard errors (SEs) to incorporate the uncertainty in the first stage predicted phenotype values, estimation of both stages was performed jointly within the generalized method of moments (GMM) framework. We additionally estimated causal RRs using multiplicative structural mean models (MSMMs) (equivalent to the multiplicative generalized method of moments [MGMM]) [22],[23] with bootstrapped SEs. When performing MSMM estimation, if the GMM estimation algorithm did not converge, then we used the Newton-Raphson algorithm. In some situations the MGMM/MSMM approach did not result in unique solutions: these are indicated in Table S4. To examine evidence of pleiotropy we derived a forest plot displaying IV estimates (and 95% CIs) derived using individual SNPs contributing to the allele score, and conducted overidentification tests of differences between these SNP-based IV estimates. All statistical analyses were performed using Stata v.13 (Stata Corp).

## Results

There were 4,835 children (594 [12.3%] with asthma) in whom the presence/absence of current asthma at 7½ y, BMI at 7 y, and data on the 32 BMI-related SNPs were recorded. 4,086 of these children also had complete data on fat mass and lean mass at 9 y. Table 1 shows characteristics of these children and the risks of current asthma at 7½ y. Mean BMI (16.2 kg/m^2^, SE = 2.0), fat mass (8.4 kg, SE = 4.9), and lean mass (24.5 kg, SE = 3.1) appeared similar in children with and without asthma, although mean BMI was higher in girls with (16.7 kg/m^2^) than without (16.2 kg/m^2^) asthma. The prevalence of atopy was much higher in children with (252 [50.3%]) than without (570 [15.8%]) asthma. The prevalence of maternal prenatal smoking was somewhat higher in children with asthma (161 [27.3%]) than without (1,013 [24.2%]), and similarly for postnatal maternal smoking (105 [18.9%] versus 686 [16.9%]). The risk of current asthma at 7½ y appeared higher for children categorized as obese (risk 0.21, 95% CI 0.14–0.31), atopic (0.31, 95% CI 0.27–0.35), and male (0.14, 95% CI 0.13–0.16). Children excluded from analyses because of missing data tended to have lower birth weight, and their mothers tended to have lower educational attainment and to smoke during and after pregnancy, compared with children included in the analyses (Table 2).

**Table 1:** Characteristics of 4,835 children with data on genetic markers, asthma, and BMI and risks of current asthma at 7½ y.

Characteristic	Number Non-Asthma/Asthma^a^	Risk (95% CI) of Current Asthma at 7½ y
**BMI (kg/m^2^) at 7 y** †	4,241/594	
Normal	3,685/502	0.12 (0.11, 0.13)
Overweight	441/62	0.12 (0.09, 0.16)
Obese	115/30	0.21 (0.14, 0.31)
**Fat mass (kg) at 9 y**	3,586/500	
≤median residual fat mass	1,840/233	0.11 (0.10, 0.13)
>median residual fat mass	1,746/267	0.13 (0.12, 0.15)
**Lean mass (kg) at 9 y** ‡	3,586/500	
≤median residual lean mass	1,807/236	0.12 (0.10, 0.13)
>median residual lean mass	1,779/264	0.13 (0.11, 0.15)
**Atopy at 7½ y (skin prick test)**	3,608/501	
No	3,038/249	0.08 (0.07, 0.09)
Yes	570/252	0.31 (0.27, 0.35)
**Gender**	4,241/594	
Male	2,107/352	0.14 (0.13, 0.16)
Female	2,134/242	0.10 (0.09, 0.12)
**Low birth weight (<2,500 g)**	4,191/587	
No	4,067/569	0.12 (0.11, 0.13)
Yes	124/18	0.13 (0.08, 0.21)
**Prenatal maternal smoking**	4,188/589	
No	3,175/428	0.12 (0.11, 0.13)
Yes	1,013/161	0.14 (0.12, 0.16)
**Postnatal maternal smoking**	4,054/555	
No	3,368/450	0.12 (0.11, 0.13)
Yes	686/105	0.13 (0.11, 0.16)
**Lower level maternal education** *	4,157/582	
No	1,927/264	0.12 (0.11, 0.14)
Yes	2,230/318	0.12 (0.11, 0.14)

a Number of children with data available.

† BMI cut points for overweight and obese defined by Cole et al. [16].

‡ Median lean mass and median fat mass were calculated and used separately for females and males.

*Educated to General Certificate of Education level (school leaving certificate at 16 y) or lower.

**Table 2:** Comparing participants included and excluded in these analyses.

Characteristic	Included (*n* = 4,835)	Excluded (*n* = 9,227)	*p*-Value* Comparing Included and Excluded
*n* (%) female	2,376 (49.1%)	4,414 (47.9%)	0.145
Mean [SD] height (m) at 9 y	1.39 [0.06]	1.40 [0.06]	0.54
*n* (%) low birth weight (<2,500 g)	142 (3.0%)	661 (7.3%)	<0.001
*n* (%) lower level of maternal education**	2,548 (53.8%)	5,517 (71.4%)	<0.001
*n* (%) prenatal maternal smoking	1,174 (24.6%)	3,319 (39.2%)	<0.001
*n* (%) postnatal maternal smoking	791 (17.2%)	1,927 (29.1%)	<0.001

Of the 14,062 participants, 8,365 had genetic data (after genetic quality control filters excluding individuals with incorrect sex assignments, minimal or excessive heterozygosity, disproportionate levels of missingness, cryptic relatedness, and non-European ancestry). 4,835 individuals had both genetic data and data on BMI and current asthma at 7–7½ y, and these individuals form the core sample for these analyses.

*Chi-squared tests and *t*-test were used as appropriate.

**General Certificate of Education level (school leaving certificate at 16 y) or lower, compared with A-level (qualification at 18 y) or degree level.

### Associations of BMI, Fat Mass, and Lean Mass with Current Asthma

Data on BMI-related SNPs, current asthma at 7½ y, and confounders were complete for 4,467 children with data on BMI at 7 y and for 3,812 children with data on fat mass, lean mass, and height at 9 y. Table 3 shows associations of BMI, fat mass, and lean mass with current asthma at 7½ and 9 y before and after stratification by gender. BMI, fat mass, and lean mass were associated with current asthma at 7½ y (adjusted RR for BMI 1.04 [95% CI 1.00–1.08] per kg/m^2^; fat mass 1.06 [95% CI 1.02–1.10] per 0.5 kg; lean mass 1.06 [95% CI 1.00–1.11] per kg). Fat mass was also associated with current asthma at 9 y (1.03 [95% CI 1.00–1.07] per 0.5 kg). Associations with BMI and lean mass appeared stronger in females than males when using current asthma at 7½ y (1.08 [95% CI 1.03–1.14] versus 0.99 [95% CI 0.94–1.05], interaction *p* = 0.02, for BMI; 1.11 [95% CI 1.03–1.20] versus 1.01 [95% CI 0.94–1.08], interaction *p* = 0.04, for lean mass), and for BMI and fat mass when using current asthma at 9 y (1.06 [95% CI 1.01–1.11] versus 0.96 [95% CI 0.91–1.01], interaction *p* = 0.005, for BMI; 1.07 [95% CI 1.02–1.13] versus 1.00 [95% CI 0.96–1.05], interaction *p* = 0.04, for fat mass). There was little evidence that the association of fat mass with current asthma at 7½ y and the association of lean mass with current asthma at 9 y differed between boys and girls (interaction *p* = 0.37 and 0.29, respectively).

**Table 3:** Associations of BMI, fat mass, and lean mass with current asthma, non-atopic asthma, and atopic asthma in children at 7½ and 9 y.

Exposure	Number Non-Asthma/Asthma/Non-Atopic Asthma/Atopic Asthma	Adjusted† RR (95% CI)			Heterogeneity *p*-Value^b^
		Current Asthma	Non-Atopic Asthma^a^	Atopic Asthma^a^	
**At 7½ y**^a^					
**BMI (kg/m^2^) at 7 y**	3,928/539/216/239	1.04 (1.00, 1.08)	1.08 (1.02, 1.14)	0.98 (0.92, 1.05)	0.03
Males	1,942/315/114/147	0.99 (0.94, 1.05)	1.06 (0.96, 1.16)	0.91 (0.83, 1.01)	
Females	1,986/224/102/92	1.08 (1.03, 1.14)	1.09 (1.01, 1.18)	1.06 (0.97, 1.16)	
*p*-Value for gender interaction‡		0.02	0.46	0.04	
**Fat mass (kg/2) at 9 y** *	3,352/460/186/206	1.06 (1.02, 1.10)	1.09 (1.03, 1.15)	1.01 (0.95, 1.07)	0.06
Males	1,634/262/96/124	1.04 (0.99, 1.10)	1.07 (0.99, 1.17)	1.00 (0.92, 1.08)	
Females	1,718/198/90/82	1.07 (1.02, 1.13)	1.10 (1.02, 1.19)	1.02 (0.92, 1.12)	
*p*-Value for gender interaction‡		0.37	0.60	0.72	
**Lean mass (kg) at 9 y** *	3,352/460/186/206	1.06 (1.00, 1.11)	1.14 (1.05, 1.24)	0.98 (0.90, 1.06)	0.01
Males	1,634/262/96/124	1.01 (0.94, 1.08)	1.09 (0.97, 1.23)	0.94 (0.85, 1.05)	
Females	1,718/198/90/82	1.11 (1.03, 1.20)	1.19 (1.07, 1.33)	1.03 (0.90, 1.16)	
*p*-Value for gender interaction‡		0.04	0.25	0.32	
**At 9 y** c					
**BMI (kg/m^2^) at 7 y**	3,377/644/257/283	1.01 (0.98, 1.05)	1.05 (0.99, 1.11)	0.96 (0.91, 1.03)	0.06
Males	1,656/374/130/180	0.96 (0.91, 1.01)	1.02 (0.94, 1.12)	0.88 (0.81, 0.97)	
Females	1,721/270/127/103	1.06 (1.01, 1.11)	1.06 (0.98, 1.14)	1.06 (0.98, 1.16)	
*p*-Value for gender interaction‡		0.005	0.41	0.005	
**Fat mass (kg/2) at 9 y** *	2,952/558/222/253	1.03 (1.00, 1.07)	1.06 (1.01, 1.12)	1.00 (0.94, 1.06)	0.10
Males	1,429/319/111/160	1.00 (0.96, 1.05)	1.04 (0.96, 1.13)	0.97 (0.90, 1.04)	
Females	1,523/239/111/93	1.07 (1.02, 1.13)	1.09 (1.01, 1.17)	1.05 (0.96, 1.14)	
*p*-Value for gender interaction‡		0.04	0.29	0.18	
**Lean mass (kg) at 9 y** *	2,952/558/222/253	1.03 (0.99, 1.08)	1.09 (1.01, 1.17)	0.99 (0.92, 1.07)	0.08
Males	1,429/319/111/160	1.01 (0.95, 1.07)	1.07 (0.96, 1.20)	0.96 (0.87, 1.05)	
Females	1,523/239/111/93	1.06 (0.99, 1.13)	1.10 (0.99, 1.22)	1.03 (0.92, 1.16)	
*p*-Value for gender interaction‡		0.29	0.65	0.37	

† Adjusted for gender, birth weight, pre- and postnatal maternal exposure to smoking, and maternal education.

a Controls were children with no current asthma at 7½ y.

b Chi-squared test comparing estimate effects for non-atopic and atopic asthma.

‡ Test for the null hypothesis that the RR in males is the same as the RR in females (likelihood ratio test).

*Based on residuals from regression models of mass measure (fat/lean mass) on height, height squared, and gender. Fat mass was divided by two so that its standard deviation was similar to that of BMI and lean mass.

c Controls were children with no current asthma at 9 y.

Adjusted associations with BMI, fat mass, and lean mass appeared stronger for non-atopic than for atopic current asthma at 7½ y (1.08 [95% CI 1.02–1.14] versus 0.98 [95% CI 0.92–1.05], heterogeneity *p* = 0.03, for BMI; 1.09 [95% CI 1.03–1.15] versus 1.01 [95% CI 0.95–1.07], heterogeneity *p* = 0.06, for fat mass; 1.14 [95% CI 1.05–1.24] versus 0.98 [95% CI 0.90–1.06], heterogeneity *p* = 0.01, for lean mass). Smaller evidence of heterogeneity was found when using current asthma at 9 y (*p* = 0.06 for BMI, 0.10 for fat mass, and 0.08 for lean mass).

Table S1 shows allele frequencies and associations of individual SNPs with BMI, fat mass, lean mass, and current asthma at age 7½ y. The strongest associations were for rs571312 (near the *MC4R* gene) (regression coefficient 0.20 kg/m^2^ per allele [95% CI 0.10–0.29] for BMI; 0.25 0.5 kg per allele [95% CI 0.13–0.36] for fat mass; 0.12 kg per allele [95% CI 0.04–0.21] for lean mass). There was little evidence for associations of individual SNPs with asthma.

### Associations of Weighted Allele Score with BMI, Fat Mass, Lean Mass, and Current Asthma

Table 4 shows that the weighted allele score was strongly associated with BMI (regression coefficient 2.22 [95% CI 1.76–2.67] kg/m^2^ per unit score, *p*<0.001), fat mass (2.88 [95% CI 2.35–3.42] 0.5 kg per unit, *p*<0.001), lean mass (1.22 [95% CI 0.80–1.64] kg per unit, *p*<0.001), current asthma at 7½ y (RR 2.56 [95% CI 1.38–4.76] per unit, *p* = 0.003), and current asthma at 9 y (RR 1.98 [95% CI 1.13–3.46] per unit, *p* = 0.02). The weighted allele score appeared more strongly associated with non-atopic than atopic asthma (RR 3.92 [95% CI 1.45–10.59] per unit versus 1.93 [95% CI 0.72–5.21] at 7½ y; 2.26 [95% CI 0.89–5.69] versus 1.49 [95% CI 0.61–3.66] at 9 y), though there was little evidence of heterogeneity (*p* = 0.32 and 0.52, respectively). We found little evidence that the weighted allele score was associated with atopy (RR 0.82 [95% CI 0.50–1.63] per unit, *p* = 0.45).

**Table 4:** Associations of the weighted allele score with BMI at age 7½ and 9 y.

Outcome	Number of Children (Total or Non-Asthma/Asthma)	Linear Regression Coefficient or RR (95% CI), *p*-Value†
**BMI (kg/m^2^) at 7 y**	4,835	2.22 (1.76, 2.67), *p* = 2.3×10^−21^
**Fat mass (kg/2) at 9 y** *	4,086	2.88 (2.35, 3.42), *p* = 2.0×10^−25^
**Lean mass (kg) at 9 y** *	4,086	1.22 (0.80, 1.64), *p* = 1.6×10^−8^
**Current asthma at 7½ y** a	4,241/594	2.56 (1.38, 4.76), *p* = 0.003
Non-atopic asthma^a^	4,241/249	3.92 (1.45, 10.59), *p* = 0.007
Atopic asthma^a^	4,241/252	1.93 (0.72, 5.21), *p* = 0.19
Heterogeneity *p*-value^b^		0.32
**Current asthma at 9 y** c	3,597/701	1.98 (1.13, 3.46), *p* = 0.016
Non-atopic asthma^c^	3,597/286	2.26 (0.89, 5.69), *p* = 0.09
Atopic asthma^c^	3,597/300	1.49 (0.61, 3.66), *p* = 0.38
Heterogeneity *p*-value^b^		0.52
Atopy at 7½ y (skin prick test)	3,553/937	0.82 (0.50, 1.63), *p* = 0.45

† Linear regression coefficient, adjusted for gender. RR for number non-asthma/asthma and number non-atopic/atopic.

*Based on residuals from regression models of mass measure (fat/lean mass) on height, height squared, and gender. Fat mass was divided by two so that its standard deviation was similar to that of BMI and lean mass.

a Controls were children with no current asthma at 7½ y.

b Chi-squared test comparing estimate effects for non-atopic and atopic asthma.

c Controls were children with no current asthma at 9 y.

**Figure 2:**
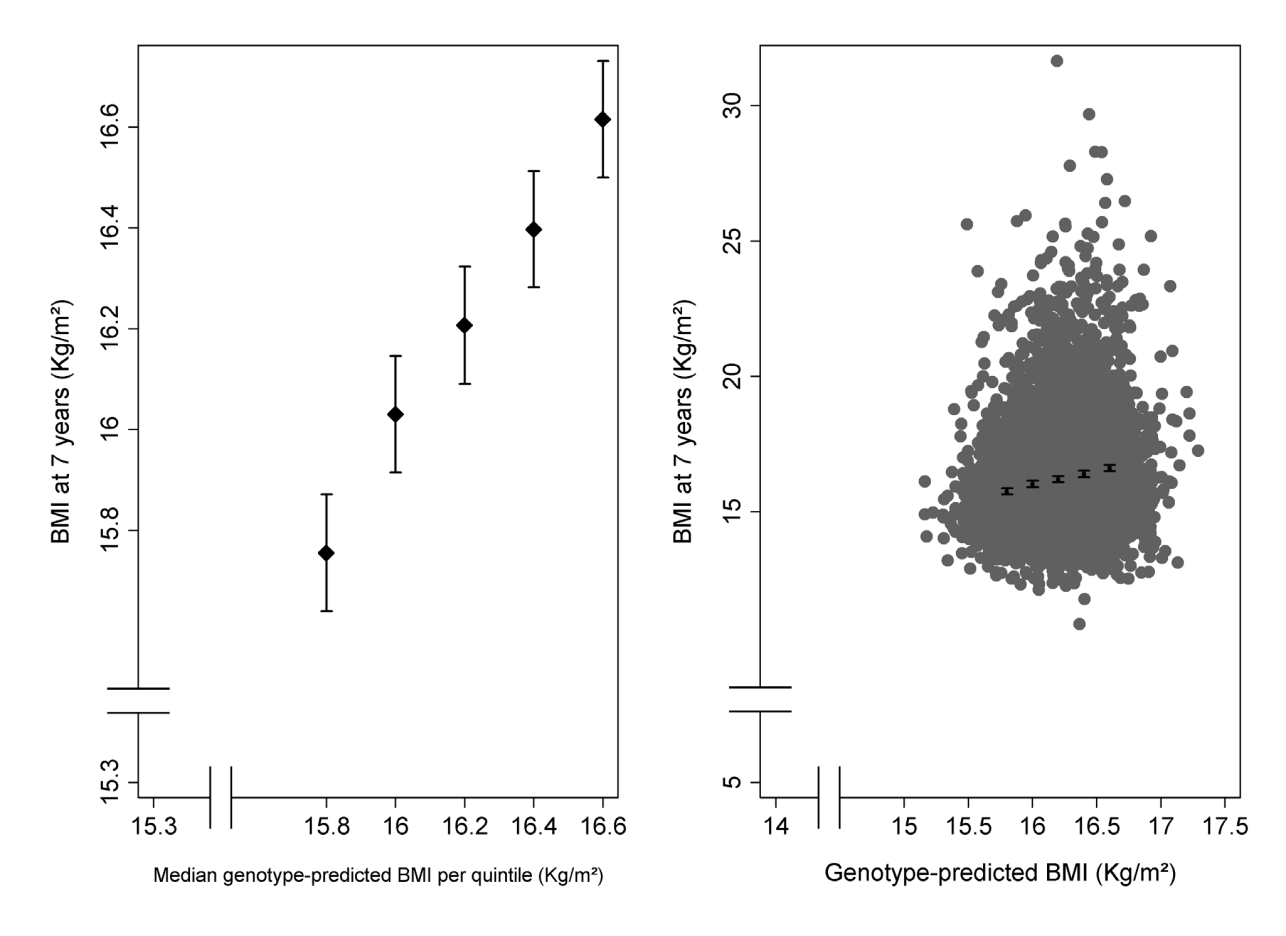
Association of BMI at 7½ y of 32 BMI-related SNPs. The left panel shows the mean BMI (with 95% CI) in groups defined by quintiles of genotype-predicted BMI. The right panel shows the same means and confidence intervals superimposed on the overall distribution of BMI plotted against genotype-predicted BMI.

Figure 2 (left panel) shows that there was a clear linear trend of mean BMI across groups defined by quintiles of genotype-predicted BMI. The right panel of Figure 2 shows the same data superimposed on the overall distribution of BMI: although there is extremely strong evidence of association, genotype explains only 2.1% of the between-child variability in BMI. As expected, we found little evidence of associations between the five confounding variables and the 32 selected SNPs (Table S2) or the weighted allele score (Table S3).

### Instrumental Variable Estimates of Causal Effects of BMI, Fat Mass, and Lean Mass on Current Asthma

**Figure 3:**
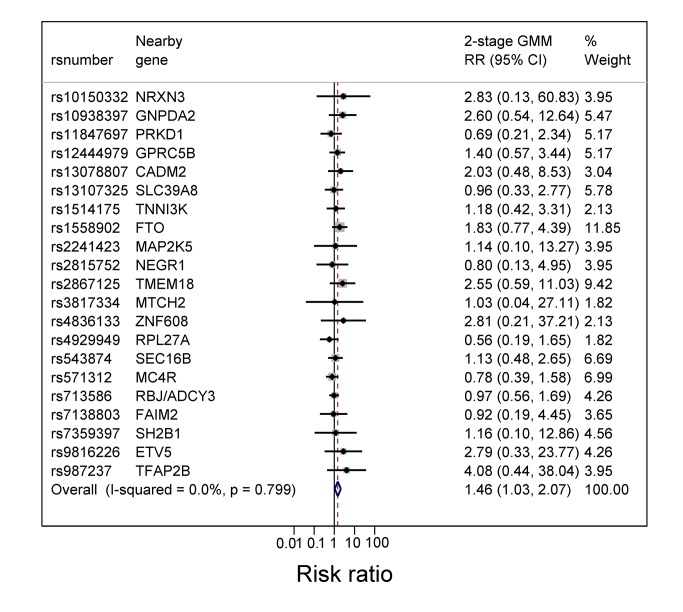
Forest plot of the individual causal effects on current asthma at 7½ y of 32 BMI-related SNPs. The SNPs are those previously reported in a meta-analysis [9] that excluded ALSPAC data. rs2287019 and rs4771122 were omitted from the forest plot because IV RRs were not estimable. Also rs10150332, rs10767664, rs10938397, rs1555543, rs206936, rs2112347, rs2287019, rs2890652, rs381029, rs4771122, rs4836133, rs9816226, and rs987237 were excluded because the corresponding SE (of the log RR) was >2.

Table 5 shows the estimated RRs for the effects of BMI, fat mass, and lean mass on current asthma and asthma subtypes, derived by using the weighted allele score as IV and using two-stage GMM estimation. The estimated causal RRs for the effects of BMI on current asthma at 7½ and 9 y were 1.55 (95% CI 1.16–2.07) and 1.38 (95% CI 1.06–1.80) per kg/m^2^, respectively. At age 7½ y this effect appeared greater for non-atopic asthma (1.90 [95% CI 1.19–3.03]) than atopic asthma (1.37 [95% CI 0.89–2.11]), though there was little evidence of heterogeneity (*p* = 0.31). The effects of BMI on asthma at age 7½ y appeared similar in girls (1.77 [95% CI 1.13–2.77]) and boys (1.40 [95% CI 0.96–2.04]) (interaction *p* = 0.43). Estimated causal RRs for the effects of fat mass and lean mass on current asthma at 9 y were 1.28 (95% CI 1.03–1.59) per 0.5 kg and 1.74 (95% CI 1.04–2.90) per kg, respectively, with little evidence for a difference between non-atopic and atopic asthma (heterogeneity *p* = 0.65 and 0.58, respectively). The estimated RR for the effect of BMI on asthma at 7½ y was 1.83 (95% CI 0.77–4.39) when using the *FTO* variant alone as an IV, and 1.50 (95% CI 1.10–2.04) when using an allele score based on the other 31 SNPs. The forest plot in Figure 3 shows that IV estimates derived using the individual BMI-related SNPs were consistent with each other. Overidentification tests of differences between these IV estimates gave no evidence against the null hypothesis of the joint validity of the multiple instruments (all *p*-values≥0.44).

**Table 5:** Instrumental variable estimates of the causal effect of BMI on current asthma, non-atopic asthma, and atopic asthma in children at age 7½ and 9 y using two-stage GMM estimator.

Exposure	Number Non-Asthma/Asthma/Non-Atopic Asthma/Atopic Asthma	Two-Stage GMM RR (95% CI)			Heterogeneity *p*-Value^a^
		Current Asthma	Non-Atopic Asthma	Atopic Asthma	
**At 7½ y**^b^					
**BMI (kg/m^2^) at 7 y**	4,241/594/249/252	1.55 (1.16, 2.07), *p* = 0.003	1.90 (1.19, 3.03), *p* = 0.008	1.37 (0.89, 2.11), *p* = 0.16	0.31
Males	2,107/352/133/158	1.40 (0.96, 2.04), *p* = 0.08‡	1.47 (0.78, 2.77), *p* = 0.23‡	1.25 (0.71, 2.20), *p* = 0.43‡	
Females	2,134/242/116/94	1.77 (1.13, 2.77), *p* = 0.01‡	2.49 (1.22, 5.06), *p* = 0.01‡	1.54 (0.78, 3.02), *p* = 0.21‡	
*p*-Value for gender interaction		0.43	0.28	0.64	
**Fat mass (kg/2) at 9 y** *	3,586/500/209/216	1.41 (1.11, 1.79), *p* = 0.006	1.73 (1.17, 2.55), *p* = 0.006	1.25 (0.86, 1.80), *p* = 0.24	0.24
**Lean mass (kg) at 9 y** *	3,586/500/209/216	2.25 (1.23, 4.11), *p* = 0.009	4.36 (1.40, 13.59), *p* = 0.01	1.69 (0.70, 4.12), *p* = 0.25	0.20
**At 9 y**^b^					
**BMI (kg/m^2^) at 7 y**	3,597/701/286/300	1.38 (1.06, 1.80), *p* = 0.02	1.44 (0.95, 2.18), *p* = 0.09	1.20 (0.81, 1.78), *p* = 0.36	0.53
Males	1,775/414/147/193	1.46 (1.00, 2.15), *p* = 0.05‡	1.38 (0.74, 2.59), *p* = 0.31‡	1.24 (0.74, 2.10), *p* = 0.41‡	
Females	1,822/287/139/107	1.29 (0.89, 1.88), *p* = 0.18‡	1.50 (0.86, 2.62), *p* = 0.16‡	1.13 (0.62, 2.05), *p* = 0.68‡	
*p*-Value for gender interaction		0.65	0.85	0.82	
**Fat mass (kg/2) at 9 y** *	3,133/602/244/267	1.28 (1.03, 1.59), *p* = 0.03	1.33 (0.95, 1.87), *p* = 0.10	1.19 (0.85, 1.65), *p* = 0.32	0.65
**Lean mass (kg) at 9 y** *	3,133/602/244/267	1.74 (1.04, 2.90), *p* = 0.03	2.05 (0.86, 4.92), *p* = 0.11	1.48 (0.69, 3.18), *p* = 0.32	0.58

a Chi-squared test comparing estimate effects for non-atopic and atopic asthma.

b Controls were children with no current asthma.

‡ Test for the null hypothesis that the RR in males is the same as the RR in females (likelihood ratio test).

*Based on residuals from regression models of mass measure (fat/lean mass) on height, height squared, and gender. Fat mass was divided by two so that its standard deviation was similar to that of BMI and lean mass.

Using the MSMM/MGMM approach, the estimated RRs were 2.34 (95% CI 1.30–4.24) per kg/m^2^ for the effect of BMI on current asthma at 7½ y, 1.75 (95% CI 1.19–2.55) per 0.5 kg for fat mass, and 1.55 (95% CI 0.99–2.44) per kg for lean mass (Table S4). However, we noted problems in estimation for some effects, because of either lack of convergence or a double solution.

Table 6 shows the observational and IV estimates of the effect of BMI at 7 y on current asthma at ages 11 to 14 y and ever doctor-diagnosed asthma at ages 7½ to 15 y, also stratified by gender and for non-atopic and atopic asthma. The results are consistent with a positive causal effect of BMI on asthma that did not decrease notably with increasing age, although the magnitudes of estimated effects at some ages were smaller than for the primary outcomes at ages 7 and 9 y reported in Table 5.

**Table 6:** Observational and instrumental variable estimates of the effect of BMI on current asthma at ages 11 to 14-diagnosed asthma at ages 7½ to 15 y in boys and girls and stratified by atopy at 7½ y.

Outcome	Observational Estimates of BMI (kg/m^2^) at 7 y on Outcome		IV Estimates of BMI (kg/m^2^) at 7 y on Outcome	
	Number Non-Asthma/Asthma	Adjusted† RR (95% CI)	Number Non-Asthma/Asthma	Two-Stage GMM RR (95% CI)
**Current asthma at 11 y** *	2,987/591	0.99 (0.95, 1.03)	3,169/633	1.27 (0.96, 1.68)
Males	1,435/350	0.95 (0.90, 1.01)	1,523/378	1.21 (0.82, 1.76)
Females	1,552/241	1.04 (0.98, 1.10)	1,646/255	1.38 (0.91, 2.08)
Non-atopic asthma	2,987/224	1.01 (0.95, 1.08)	3,169/241	1.51 (0.93, 2.46)
Atopic asthma	2,987/267	0.95 (0.89, 1.01)	3,169/282	1.09 (0.72, 1.64)
**Current asthma at 13 y** *	3,151/515	1.01 (0.97, 1.05)	3,348/545	1.48 (1.07, 2.04)
Males	1,527/298	0.98 (0.92, 1.04)	1,628/317	1.20 (0.76, 1.90)
Females	1,624/217	1.05 (0.99, 1.11)	1,720/228	1.86 (1.18, 2.94)
Non-atopic asthma	3,151/203	1.04 (0.97, 1.11)	3,348/217	2.66 (1.47, 4.82)
Atopic asthma	3,151/227	0.94 (0.87, 1.01)	3,348/239	1.06 (0.67, 1.66)
**Current asthma at 14 y** *	2,728/537	0.97 (0.93, 1.02)	2,886/570	1.29 (0.92, 1.80)
Males	1,320/296	0.94 (0.88, 1.01)	1,392/318	1.36 (0.85, 2.19)
Females	1,408/241	1.00 (0.95, 1.06)	1,494/252	1.19 (0.74, 1.90)
Non-atopic asthma	2,728/213	0.99 (0.93, 1.06)	2,886/227	1.91 (1.07, 3.42)
Atopic asthma	2,728/235	0.95 (0.88, 1.02)	2,886/247	0.92 (0.57, 1.49)
**Ever DDA at 7½ y** *	3,600/867	1.03 (1.00, 1.06)	3,877/958	1.43 (1.15, 1.78)
Males	1,769/488	0.99 (0.95, 1.04)	1,911/548	1.38 (1.03, 1.86)
Females	1,831/379	NA	1,966/410	1.47 (1.07, 2.03)
Non-atopic asthma	3,600/436	1.06 (1.02, 1.10)	3,877/494	1.78 (1.26, 2.50)
Atopic asthma	3,600/298	0.97 (0.91, 1.03)	3,877/315	1.29 (0.86, 1.93)
**Ever DDA at 11 y**	3,147/805	1.03 (1.00, 1.06)	3,342/867	1.40 (1.12, 1.75)
Males	1,530/469	0.99 (0.94, 1.03)	1,625/509	1.36 (0.99, 1.85)
Females	1,617/336	1.06 (1.02, 1.10)	1,717/358	1.45 (1.06, 2.00)
Non-atopic asthma	3,147/390	1.06 (1.02, 1.11)	3,342/426	1.83 (1.29, 2.60)
Atopic asthma	3,147/292	0.96 (0.90, 1.02)	3,342/305	1.17 (0.80, 1.72)
**Ever DDA at 14 y**	2,882/810	1.01 (0.98, 1.04)	3,047/868	1.36 (1.07, 1.72)
Males	1,406/445	0.98 (0.93, 1.02)	1,484/483	1.38 (1.00, 1.91)
Females	1,476/365	1.04 (1.00, 1.08)	1,563/385	1.32 (0.94, 1.84)
Non-atopic asthma	2,882/402	1.05 (1.00, 1.09)	3,047/435	1.83 (1.25, 2.66)
Atopic asthma	2,882/291	0.95 (0.89, 1.01)	3,047/306	1.05 (0.70, 1.59)
**Ever DDA at 15 y**	2,076/594	1.05 (1.02, 1.09)	2,214/635	1.43 (1.10, 1.84)
Males	985/304	1.02 (0.96, 1.07)	1,046/332	1.36 (0.92, 2.01)
Females	1,091/290	NA	1,168/303	1.48 (1.06, 2.07)
Non-atopic asthma	2,076/298	NA	2,214/324	1.68 (1.14, 2.46)
Atopic asthma	2,076/213	0.98 (0.91, 1.05)	2,214/223	1.25 (0.78, 1.99)

† Adjusted for gender, birth weight, pre- and postnatal maternal exposure to smoking, and maternal education.

*Derived using ever doctor-diagnosed asthma (available at 7½, 11, 14, and 15 y) and parent-reported current asthma or wheezing or treatment in last 12 mo.

DDA, doctor-diagnosed asthma; NA, not available.

## Discussion

### Main Findings

Based on a large, population-based birth cohort study we confirmed a positive association between BMI and asthma in mid-childhood and, using Mendelian randomization analyses based on 32 BMI-associated SNPs, found strong evidence that this association arose from a causal effect of BMI on asthma. The effect of BMI appeared stronger for non-atopic than atopic asthma, though evidence for interaction was weak. We found evidence that higher fat mass and lean mass increase the risk of asthma in mid-childhood. Effects persisted to age 15 y.

As an illustration of the possible public health impact of these findings, the prevalence of asthma in UK children was estimated to increase from around 6% to over 20% between 1975 and 2000 [24]. Contemporaneous increases in the BMI of a sample of 11- to 12-y-old children in the UK [25] were 1.54 kg/m^2^ in boys and 1.62 kg/m^2^ in girls. If these increases also applied in younger children, they could account for up to a doubling of asthma prevalence (1.7-fold in boys [1.40^1.54^] and 2.5-fold in girls [1.77^1.62^]) based on our IV estimates.

### Results in the Context of the Existing Literature

Our results are consistent with reports from observational studies of the association between BMI and asthma in children. The strongest evidence has come from prospective cohort studies, which have mostly found positive associations of BMI in childhood with incident asthma, summarized in a recent systematic review [26]. Similar associations between BMI and asthma have been reported in adults, with effect sizes similar to our IV estimates [27]. The origins of this association are not well understood. A plausible explanation is that obesity is associated with systemic inflammation, which may give rise to airway inflammation and asthma. There is evidence that adipocytes are a source of pro-inflammatory cytokines [28] but little evidence that systemic inflammation in obesity is directly associated with airway inflammation [29]. Another possible link would be through promotion of allergic inflammation by adipokine effects on the immune system, but, like us, others have reported stronger associations of obesity with non-atopic asthma [30], and we found no evidence that obesity is associated with atopy in mid-childhood. A specific asthma-obesity phenotype has been suggested in both adults and children [31], which may be associated with increased asthma severity. There is evidence that obesity in established asthma is associated with poor asthma control, increased exacerbations, and suboptimal response to glucocorticoids [32]. Poor response to steroids may be associated with neutrophil-predominant airway inflammation [33], consistent with our finding of a stronger association with non-atopic asthma.

Studies in adults (systematically reviewed by Beuther and Sutherland [27]) have reported positive associations of overweight and incident asthma, with increasing effects in obese compared with overweight individuals. Consistent results are reported from childhood studies that prospectively ascertained BMI and incident asthma: two recent systematic reviews [26],[34] have synthesized these results. Chen et al. [34] analyzed six studies of prospective cohorts of children aged 5–18 y that investigated associations of BMI with asthma incidence, reporting increased odds of asthma for overweight and obese children and a dose-dependent effect of BMI. Chen et al. specifically addressed gender stratification and reported a greater effect size in males than in females. Egan et al. [26] identified six studies meeting inclusion criteria that had data on BMI in children <18 y and incident asthma at least 12 mo after BMI was measured. There was evidence of a positive association between overweight and asthma, but inconsistent evidence of sexual dimorphism in this relationship, similar to the findings in adult prospective studies. Our study did not provide strong evidence for sex stratification of the association between genetically predicted BMI and asthma. Inconsistent findings in the literature could reflect differences in definitions and categorization of exposures and outcome in different studies but may also relate to the timing of studies in relation to other influences on body composition, some of which, such as age at menarche, might themselves be under genetic influence. For example, in the 1958 British National Child Development Study birth cohort, females with early menarche were more likely to be overweight [35], and although age at menarche did not explain the association of obesity with asthma in this study, both early menarche and obesity were independently associated with persistence of asthma symptoms in the Tucson Children's Respiratory Study, which followed children through adolescence [36]. Therefore, influences on body composition that are unrelated to genetic prediction of BMI could account for between-sex differences in associations.

The majority of studies of body mass and asthma have focused on obesity and the inflammatory mechanisms through which fat mass could induce the development of asthma. We found evidence that both higher fat mass and higher lean mass increase the risk of asthma. Each was measured in kilograms, but the standard deviation of fat mass residuals was more than twice that of lean mass residuals, and so we expressed the relationship between fat mass and asthma as per 0.5 kg. Therefore, the odds ratios for fat mass and lean mass are not directly comparable: their public health implications for asthma risk will depend on the extent to which they are affected by interventions such as dietary change or increased exercise. Interventions to reduce BMI in obese children and adolescents have been multifaceted (diet and physical activity) and aimed at preserving lean mass while reducing BMI and fat mass. The effects of such interventions have been demonstrated in randomized controlled trials [37],[38]. Therefore, public health interventions are likely to have a greater impact on fat mass than lean mass, with consequent differences in the impact of these interventions on absolute asthma risk.

For example, Knöpfli et al. [38] quantified the effects of a multidisciplinary inpatient intervention on body composition in 130 severely obese children, and observed reductions of 4.8 kg/m^2^ in BMI, 8.2 kg in fat mass, and 2.8 kg in fat-free (lean) mass. Based on the IV estimates in Table 5, corresponding RRs for asthma are 1.55^−4.8^ = 0.12 for BMI (88% risk reduction), 1.41^−16.4^ = 0.004 for fat mass (99% risk reduction), and 2.25^−2.8^ = 0.10 for lean mass (90% risk reduction). Similarly, Parks et al. [37] quantified change in body composition during a weight loss trial in 61 obese adolescents, and the corresponding RRs for asthma based on our IV estimates are 1.55^−3.2^ = 0.24 for BMI (76% risk reduction), 1.41^−16.0^ = 0.004 for fat mass (99% risk reduction), and 2.25^−2.2^ = 0.17 for lean mass only among girls (83% risk reduction only among girls).

There is some evidence that body composition influences asthma other than through obesity-related inflammatory mechanisms. In a cross-sectional analysis, Sood et al. [39] reported that lean mass, particularly truncal lean mass, was a better predictor of asthma than fat mass in females, suggesting an effect of ectopic fat in muscle and viscera, possibly through ready release of inflammatory mediators from these sources into the systemic circulation [40]. A study of obese adults with asthma has reported sexual dimorphism and an association of lean mass with neutrophilic airway inflammation in obese females with asthma [41]. Further elucidation of these effects will require quantification of both fat and lean mass, and their relative distribution, in future studies—particularly intervention studies—of body mass and asthma.

### Strengths and Limitations

The association between BMI and asthma could be underpinned by shared heritability. Our analyses assumed that the effects of BMI-related SNPs on asthma are mediated through their effect on BMI, but we cannot discount the possibility of genetic pleiotropy. Twin studies suggest that a proportion of the covariation between obesity and asthma is explained by shared genetic factors [42],[43]. Genome-wide linkage studies have identified overlapping regions of the genome associated with both asthma and obesity [44], but no genetic variants associated with obesity and asthma have been consistently identified [45],[46]. It is conceivable that part of the heritability is explained by non-coding variation in DNA, such as methylation [47] or other epigenetic effects. Causal effect estimates may have been inflated by pleiotropy, but we found little evidence for this.

Mendelian randomization is a powerful approach to estimating the causal effects of modifiable exposures on disease outcomes in observational studies, because genetic variants do not change in response to disease and are generally unrelated to confounding factors. Our IV analyses used an allele score based on 32 SNPs whose effect on BMI was estimated in large genome-wide association studies independent of our dataset. Nonetheless, known genetic variants explain only a small proportion of population variation in BMI, so the IV analyses that underpin Mendelian randomization studies may require large sample sizes. We found larger RRs using genetically predicted BMI than using observed BMI, which was based on a single measurement. Differences between observational associations and causal effects can arise through confounding, although we think such effects would tend to bias observational associations upwards rather than downwards. The most plausible explanation for the observational association being smaller is that IV genetically predicted BMI measures lifetime exposure: changes in BMI over time would likely attenuate observed associations.

Strengths of this study include the large size of the cohort, the availability of DNA for genotyping, the ability to classify asthma as atopic or non-atopic based on skin prick testing, and the availability of fat mass and lean mass measured using DXA: BMI may not be a good proxy for body fatness in children [48]. We used reported doctor-diagnosed asthma together with reported symptoms or asthma treatment in the 12 mo prior to 7½ y as the primary outcome: we have previously found this to correlate with other measures of asthma phenotype [49]. The long-term nature of the cohort meant that we could repeat analyses for asthma up to age 15 y. Substantial loss to follow-up is inevitable in long-term cohort studies: the subset of participants analyzed in this study represents less than half of the original cohort, and more socially disadvantaged participants were more likely to be lost (Table 2). We do not believe that loss to follow-up is likely to have biased the estimated effects of BMI reported here, because missingness is predominantly in the outcome variable and is unlikely to be related to genotype [50].

### Conclusion

In conclusion, possible environmental influences on the development of asthma in childhood have been extensively investigated in epidemiological studies, but few of these provide strong evidence for causality. We used Mendelian randomization based on a weighted allele score to estimate the effect of higher BMI in mid-childhood in increasing the risk of childhood asthma. This effect could help explain some of the increase in asthma risk toward the end of the 20th century, although the continued rise in obesity but with a slowing in the rise in asthma prevalence in some countries implies that other non-BMI-related factors are also likely to be important. Our study illustrates the potential utility of using genetic instruments to investigate causal effects in observational studies, which may act as a stimulus to targeted interventions.

## Supporting Information

Table S1 **Associations of individual BMI-related SNPs with BMI, fat mass, lean mass, and asthma.**
(DOC)

Table S2 ***p*** **-Values for the individual SNP associations with confounding factors.**
(DOC)

Table S3 **Associations of potential confounding factors with BMI/fat mass/lean mass and the weighted allele score.**
(DOC)

Table S4 **Instrumental variable estimates of the causal effect of BMI on asthma in all, atopic, and non-atopic children at age 7½ y using a MSMM/MGMM estimator.**
(DOC)

## Abbreviations

ALSPAC: Avon Longitudinal Study of Parents and Children
BMI: body mass index
DXA: dual X-ray emission absorptiometry
GMM: generalized method of moments
IV: instrumental variable
MGMM: multiplicative generalized method of moments
MSMM: multiplicative structural mean model
RR: risk ratio
SE: standard error
SNP: single nucleotide polymorphism
