# Supplementary material for: Effects of BMI, Fat Mass, and Lean Mass on Asthma in Childhood: A Mendelian Randomization Study
Source: PLoS Med. 2014 Jul 1;11(7):e1001669. doi: 10.1371/journal.pmed.1001669 (PMC4077660; doi:10.1371/journal.pmed.1001669)
Supplement: Table S1 — Associations of individual BMI-related SNPs with BMI, fat mass, lean mass, and asthma. (DOC) [file pmed.1001669.s001.doc]

**Table S1. Associations of individual BMI-related SNPs with BMI, fat mass, lean mass and asthma. The number analyzed was 4835 for BMI and asthma and 4086 for fat/lean mass.**

|  |  |  |  |  |  | **Association with BMI^c^ (kg/m2)** | | **Association with fat mass^e^ (kg/2)** | | **Association with lean mass^e^(kg)** | | **Association with current asthma^c^ at 7.5 years** | |
| --- | --- | --- | --- | --- | --- | --- | --- | --- | --- | --- | --- | --- | --- |
| **SNP** | **Nearby gene** | **Chr** | **Alleles^a^** | **RAF^b^** | **R^2^** | **Per-allele change^d^ (95% CI)** | **P-value** | **Per-allele change^d^ (95% CI)** | **P-value** | **Per-allele change^d^ (95% CI)** | **P-value** | **RR per allele (95%CI)** | **P-value** |
| rs2815752 | *NEGR1* | 1 | A/G | 0.6 | 1.00 | 0.06 (-0.02,0.14) | 0.14 | 0.10 (0.00,0.19) | 0.042 | 0.05 (-0.03,0.12) | 0.2 | 0.98 (0.88,1.10) | 0.76 |
| rs1514175 | *TNNI3K* | 1 | A/G | 0.43 | 1.00 | 0.10 (0.02,0.18) | 0.011 | 0.06 (-0.03,0.16) | 0.19 | 0.08 (0.01,0.16) | 0.024 | 1.02 (0.91,1.13) | 0.74 |
| rs1555543 | *PTBP2* | 1 | C/A | 0.59 | 1.00 | 0.01 (-0.07,0.09) | 0.78 | 0.01 (-0.09,0.10) | 0.88 | 0.04 (-0.03,0.12) | 0.26 | 1.01 (0.91,1.13) | 0.85 |
| rs543874 | *SEC16B* | 1 | G/A | 0.21 | 1.00 | 0.15 (0.06,0.25) | 0.002 | 0.18 (0.07,0.29) | 0.002 | 0.06 (-0.03,0.15) | 0.18 | 1.01 (0.89,1.15) | 0.85 |
| rs2867125 | *TMEM18* | 2 | C/T | 0.83 | 1.00 | 0.12 (0.02,0.23) | 0.02 | 0.18 (0.06,0.31) | 0.004 | 0.14 (0.04,0.24) | 0.004 | 1.12 (0.97,1.30) | 0.14 |
| rs713586 | *RBJ/*  *ADCY3* | 2 | C/T | 0.49 | 1.00 | 0.19 (0.11,0.27) | 1.7×10^-6^ | 0.26 (0.17,0.35) | 1.4×10^-5^ | 0.06 (-0.01,0.13) | 0.12 | 1.00 (0.90,1.11) | 0.99 |
| rs887912 | *FANCL* | 2 | T/C | 0.29 | 1.00 | -0.01 (-0.09,0.08) | 0.9 | 0.03 (-0.07,0.13) | 0.6 | -0.02 (-0.10,0.06) | 0.63 | 1.06 (0.94,1.19) | 0.35 |
| rs2890652 | *LRP1B* | 2 | C/T | 0.17 | 0.99 | -0.03 (-0.14,0.07) | 0.54 | -0.02 (-0.14,0.10) | 0.75 | 0.07 (-0.03,0.16) | 0.17 | 0.97 (0.84,1.12) | 0.72 |
| rs13078807 | *CADM2* | 3 | G/A | 0.2 | 1.00 | 0.11 (0.01,0.21) | 0.033 | 0.03 (-0.09,0.15) | 0.6 | 0.03 (-0.06,0.13) | 0.45 | 1.07 (0.94,1.22) | 0.28 |
| rs9816226 | *ETV5* | 3 | T/A | 0.83 | 0.96 | 0.08 (-0.02,0.19) | 0.13 | 0.10 (-0.03,0.22) | 0.13 | -0.00 (-0.10,0.10) | 0.97 | 1.09 (0.94,1.26) | 0.25 |
| rs10938397 | *GNPDA2* | 4 | G/A | 0.43 | 0.99 | 0.08 (0.00,0.16) | 0.039 | 0.08 (-0.02,0.17) | 0.11 | 0.02 (-0.06,0.09) | 0.66 | 1.08 (0.97,1.20) | 0.16 |
| rs13107325 | *SLC39A8* | 4 | T/C | 0.08 | 1.00 | 0.20 (0.04,0.35) | 0.011 | 0.30 (0.12,0.49) | 0.001 | 0.19 (0.05,0.33) | 0.008 | 0.99 (0.81,1.22) | 0.96 |

**Table S1 (continued)**

|  |  |  |  |  |  | **Association with BMI^c^ (kg/m2)** | | **Association with fat mass^e^ (kg/2)** | | **Association with lean mass^e^(kg)** | | **Association with current asthma^c^ at 7.5 years** | |
| --- | --- | --- | --- | --- | --- | --- | --- | --- | --- | --- | --- | --- | --- |
| **SNP** | **Nearby gene** | **Chr** | **Alleles^a^** | **RAF^b^** | **R^2^** | **Per-allele change^d^ (95% CI)** | **P-value** | **Per-allele change^d^ (95% CI)** | **P-value** | **Per-allele change^d^ (95% CI)** | **P-value** | **RR per allele (95%CI)** | **P-value** |
| rs2112347 | *FLJ35779*  *HMGCR* | 5 | T/G | 0.64 | 0.99 | 0.03 (-0.05,0.12) | 0.41 | -0.00 (-0.10,0.10) | 0.97 | -0.01 (-0.08,0.07) | 0.84 | 1.05 (0.94,1.17) | 0.43 |
| rs4836133 | *ZNF608* | 5 | A/C | 0.48 | 0.94 | 0.05 (-0.03,0.13) | 0.2 | 0.02 (-0.08,0.11) | 0.71 | 0.09 (0.01,0.16) | 0.021 | 1.06 (0.95,1.18) | 0.31 |
| rs206936 | *HMGA1* | 6 | G/A | 0.19 | 0.99 | 0.02 (-0.08,0.12) | 0.68 | -0.04 (-0.16,0.07) | 0.46 | 0.03 (-0.06,0.12) | 0.49 | 1.03 (0.90,1.17) | 0.69 |
| rs987237 | *TFAP2B* | 6 | G/A | 0.18 | 1.00 | 0.09 (-0.01,0.19) | 0.079 | 0.16 (0.04,0.28) | 0.008 | 0.07 (-0.03,0.16) | 0.17 | 1.12 (0.98,1.28) | 0.084 |
| rs10968576 | *LRRN6C* | 9 | G/A | 0.32 | 1.00 | 0.02 (-0.06,0.11) | 0.6 | 0.00 (-0.10,0.10) | 0.93 | 0.01 (-0.07,0.09) | 0.79 | 0.97 (0.87,1.09) | 0.66 |
| rs4929949 | *RPL27A* | 11 | C/T | 0.54 | 0.97 | 0.11 (0.03,0.19) | 0.009 | 0.04 (-0.05,0.14) | 0.39 | 0.04 (-0.04,0.11) | 0.31 | 0.95 (0.85,1.05) | 0.32 |
| rs10767664 | *BDNF* | 11 | A/T | 0.79 | 1.00 | 0.03 (-0.07,0.12) | 0.61 | 0.04 (-0.07,0.16) | 0.49 | 0.07 (-0.02,0.16) | 0.13 | 1.09 (0.95,1.24) | 0.23 |
| rs3817334 | *MTCH2* | 11 | T/C | 0.4 | 1.00 | 0.03 (-0.04,0.11) | 0.39 | 0.10 (0.01,0.20) | 0.035 | 0.02 (-0.05,0.10) | 0.56 | 1.00 (0.90,1.11) | 0.98 |
| rs7138803 | *FAIM2* | 12 | A/G | 0.36 | 1.00 | 0.07 (-0.01,0.15) | 0.088 | 0.11 (0.01,0.21) | 0.03 | 0.01 (-0.07,0.08) | 0.85 | 0.99 (0.89,1.11) | 0.9 |
| rs4771122 | *MTIF3* | 13 | G/A | 0.24 | 0.93 | -0.00 (-0.10,0.09) | 0.96 | 0.01 (-0.10,0.13) | 0.82 | -0.04 (-0.13,0.04) | 0.32 | 1.07 (0.94,1.21) | 0.33 |
| rs11847697 | *PRKD1* | 14 | T/C | 0.05 | 0.97 | 0.25 (0.06,0.44) | 0.009 | 0.22 (-0.01,0.44) | 0.059 | 0.15 (-0.02,0.33) | 0.084 | 0.90 (0.69,1.18) | 0.46 |
| rs10150332 | *NRXN3* | 14 | C/T | 0.21 | 1.00 | 0.05 (-0.05,0.14) | 0.35 | 0.05 (-0.06,0.16) | 0.39 | 0.02 (-0.07,0.11) | 0.72 | 1.07 (0.94,1.21) | 0.32 |
| rs2241423 | *MAP2K5* | 15 | G/A | 0.79 | 1.00 | 0.05 (-0.04,0.15) | 0.29 | 0.02 (-0.10,0.13) | 0.75 | 0.05 (-0.04,0.14) | 0.25 | 1.01 (0.89,1.15) | 0.90 |

**Table S1(continued)**

|  |  |  |  |  |  | **Association with BMI^c^ (kg/m2)** | | **Association with fat mass^e^ (kg/2)** | | **Association with lean mass^e^(kg)** | | **Association with current asthma^c^ at 7.5 years** | |
| --- | --- | --- | --- | --- | --- | --- | --- | --- | --- | --- | --- | --- | --- |
| **SNP** | **Nearby gene** | **Chr** | **Alleles^a^** | **RAF^b^** | **R^2^** | **Per-allele change^d^ (95% CI)** | **P-value** | **Per-allele change^d^ (95% CI)** | **P-value** | **Per-allele change^d^ (95% CI)** | **P-value** | **RR per allele (95%CI)** | **P-value** |
| rs12444979 | *GPRC5B* | 16 | C/T | 0.86 | 1.00 | 0.18 (0.06,0.29) | 0.002 | 0.15 (0.01,0.28) | 0.031 | 0.10 (-0.01,0.20) | 0.072 | 1.07 (0.91,1.26) | 0.40 |
| rs7359397 | *SH2B1* | 16 | T/C | 0.41 | 1.00 | 0.05 (-0.03,0.13) | 0.24 | 0.12 (0.03,0.22) | 0.01 | 0.00 (-0.07,0.08) | 0.93 | 1.00 (0.90,1.11) | 0.99 |
| rs1558902 | *FTO* | 16 | A/T | 0.4 | 1.00 | 0.13 (0.05,0.21) | 0.001 | 0.23 (0.14,0.33) | 2.0×10^-6^ | 0.04 (-0.03,0.11) | 0.29 | 1.09 (0.98,1.21) | 0.12 |
| rs571312 | *MC4R* | 18 | A/C | 0.24 | 1.00 | 0.20 (0.10,0.29) | 4.0×10^-5^ | 0.25 (0.13,0.36) | 1.4×10^-5^ | 0.12 (0.04,0.21) | 0.005 | 0.95 (0.83,1.08) | 0.40 |
| rs29941 | *KCTD15* | 19 | G/A | 0.68 | 1.00 | 0.05 (-0.04,0.13) | 0.28 | 0.04 (-0.06,0.14) | 0.46 | 0.05 (-0.03,0.12) | 0.25 | 0.92 (0.82,1.03) | 0.16 |
| rs2287019 | *QPCTL*  *GIPR* | 19 | C/T | 0.81 | 1.00 | 0.01 (-0.09,0.11) | 0.87 | 0.00 (-0.12,0.12) | 0.98 | 0.04 (-0.06,0.13) | 0.45 | 1.17 (1.01,1.35) | 0.038 |
| rs3810291 | *TMEM160* | 19 | A/G | 0.66 | 0.77 | 0.03 (-0.06,0.13) | 0.48 | 0.13 (0.02,0.24) | 0.021 | 0.03 (-0.05,0.12) | 0.46 | 1.11 (0.97,1.26) | 0.13 |

^a^ BMI increasing allele / BMI decreasing allele

^b^ BMI increasing allele frequency

^c^ Adjusted for gender only

^d^ Chi-squared test with 2 df from co-dominant or additive model

^e^ Based on residuals from regression models of mass measure (fat/lean mass) on height, height squared, and gender
