# Supplementary material for: Effects of BMI, Fat Mass, and Lean Mass on Asthma in Childhood: A Mendelian Randomization Study
Source: PLoS Med. 2014 Jul 1;11(7):e1001669. doi: 10.1371/journal.pmed.1001669 (PMC4077660; doi:10.1371/journal.pmed.1001669)
Supplement: Table S2 — p -Values for the individual SNP associations with confounding factors. (DOC) [file pmed.1001669.s002.doc]

**Table S2. P-values for the individual SNP association with confounding factors**

| **SNP** | **Nearby gene** | **Females** | **Low Birthweight (<2500 g)** | **Prenatal maternal smoking** | **Posnatal maternal smoking** | **Maternal education*** |
| --- | --- | --- | --- | --- | --- | --- |
| rs2815752 | *NEGR1* | 0.78 | 0.45 | 0.65 | 0.49 | 0.75 |
| rs1514175 | *TNNI3K* | 0.65 | 0.69 | 0.09 | 0.04 | 0.001 |
| rs1555543 | *PTBP2* | 0.64 | 0.003 | 0.83 | 0.78 | 0.08 |
| rs543874 | *SEC16B* | 0.16 | 0.46 | 0.24 | 0.30 | 0.61 |
| rs2867125 | *TMEM18* | 0.48 | 0.12 | 0.90 | 0.35 | 0.70 |
| rs713586 | *RBJ/ADCY3/POMC* | 0.25 | 0.99 | 0.14 | 0.38 | 0.93 |
| rs887912 | *FANCL* | 0.02 | 0.18 | 0.55 | 0.74 | 0.96 |
| rs2890652 | *LRP1B* | 0.75 | 0.40 | 0.11 | 0.47 | 0.60 |
| rs13078807 | *CADM2* | 0.55 | 0.49 | 0.02 | 0.01 | 0.44 |
| rs9816226 | *ETV5* | 0.55 | 0.69 | 0.39 | 0.88 | 0.57 |
| rs10938397 | *GNPDA2* | 0.61 | 0.68 | 0.82 | 0.85 | 0.86 |
| rs13107325 | *SLC39A8* | 0.87 | 0.88 | 0.74 | 0.92 | 0.28 |
| rs2112347 | *FLJ35779/HMGCR* | 0.61 | 0.58 | 0.88 | 0.10 | 0.12 |
| rs4836133 | *ZNF608* | 0.71 | 0.72 | 0.15 | 0.80 | 0.46 |
| rs206936 | *HMGA1* | 0.24 | 0.84 | 0.58 | 0.19 | 0.15 |
| rs987237 | *TFAP2B* | 0.20 | 0.93 | 0.99 | 0.30 | 0.39 |
| rs10968576 | *LRRN6C* | 0.57 | 0.27 | 0.84 | 0.16 | 0.15 |
| rs4929949 | *RPL27A* | 0.08 | 0.57 | 0.51 | 0.29 | 0.38 |
| rs10767664 | *BDNF* | 0.54 | 0.39 | 0.65 | 0.91 | 0.07 |
| rs3817334 | *MTCH2* | 0.36 | 0.46 | 0.64 | 0.94 | 0.84 |
| rs7138803 | *FAIM2* | 0.81 | 0.05 | 0.83 | 0.29 | 0.20 |
| rs4771122 | *MTIF3* | 0.09 | 0.05 | 0.21 | 0.08 | 0.20 |
| rs11847697 | *PRKD1* | 0.14 | 0.24 | 0.59 | 0.76 | 0.41 |
| rs10150332 | *NRXN3* | 0.01 | 0.84 | 0.13 | 0.83 | 0.59 |
| rs2241423 | *MAP2K5* | 0.65 | 0.42 | 0.26 | 0.43 | 0.96 |
| rs12444979 | *GPRC5B* | 0.12 | 0.04 | 0.65 | 0.23 | 0.75 |
| rs7359397 | *SH2B1* | 0.19 | 0.63 | 1.00 | 0.33 | 0.40 |
| rs1558902 | *FTO* | 0.38 | 0.73 | 0.86 | 0.46 | 0.92 |
| rs571312 | *MC4R* | 0.04 | 0.63 | 0.43 | 0.48 | 0.66 |
| rs29941 | *KCTD15* | 0.63 | 0.53 | 0.15 | 0.36 | 0.66 |
| rs2287019 | *QPCTL/GIPR* | 0.38 | 0.11 | 0.28 | 0.32 | 0.07 |
| rs3810291 | *TMEM160* | 0.36 | 0.67 | 0.29 | 0.53 | 0.84 |

* GCE level (school leaving certificate at 16 years) or lower, compared with A-level (qualification at 18 years) or degree level

Note: These p-values have not been corrected for multiple testing. Under Bonferroni-correction the cut-off for significance is alpha= 0.05/32=0.002.
