# Supplementary material for: Effects of BMI, Fat Mass, and Lean Mass on Asthma in Childhood: A Mendelian Randomization Study
Source: PLoS Med. 2014 Jul 1;11(7):e1001669. doi: 10.1371/journal.pmed.1001669 (PMC4077660; doi:10.1371/journal.pmed.1001669)
Supplement: Table S3 — Associations of potential confounding factors with BMI/fat mass/lean mass and the weighted allele score. (DOC) [file pmed.1001669.s003.doc]

**Table S3. Associations of potential confounding factors with BMI/fat mass/lean mass and the weighted allelic score**

| **Confounders** | **Mean difference**  **(95% CI) for**  **BMI at 7 years** | **Mean difference**  **(95% CI) for**  **Fat mass at 9 years** | **Mean difference**  **(95% CI) for**  **Lean mass at 9 years** | **Linear coefficient**  **(95% CI) for weighted allelic score** |
| --- | --- | --- | --- | --- |
| Females | 0.22 (0.11,0.33) | -0.00 (-0.14,0.13) | 0.00 (-0.10,0.11) | -0.00 (-0.01,0.00) |
| Low Birthweight (<2500 g) | -0.69 (-1.02,-0.36) | -0.12 (-0.51,0.27) | -0.56 (-0.86,-0.26) | -0.01 (-0.03,0.01) |
| Prenatal maternal smoking | 0.38 (0.26,0.51) | 0.40 (0.24,0.55) | 0.20 (0.08,0.32) | 0.00 (-0.01,0.01) |
| Posnatal maternal smoking | 0.36 (0.21,0.51) | 0.46 (0.28,0.64) | 0.17 (0.03,0.31) | -0.00 (-0.01,0.01) |
| Maternal education* | 0.08 (-0.04,0.19) | 0.32 (0.19,0.45) | 0.04 (-0.07,0.14) | -0.00 (-0.01,0.00) |

* GCE level (school leaving certificate at 16 years) or lower, compared with A-level (qualification at 18 years) or degree level
