# Supplementary material for: Effects of BMI, Fat Mass, and Lean Mass on Asthma in Childhood: A Mendelian Randomization Study
Source: PLoS Med. 2014 Jul 1;11(7):e1001669. doi: 10.1371/journal.pmed.1001669 (PMC4077660; doi:10.1371/journal.pmed.1001669)
Supplement: Table S4 — Instrumental variable estimates of the causal effect of BMI on asthma in all, atopic, and non-atopic children at age 7½ y using a MSMM/MGMM estimator. (DOC) [file pmed.1001669.s004.doc]

**Table S4. Instrumental variable estimates of the causal effect of BMI on asthma in all, atopic, and non-atopic children at age 7½ using a MSMM/MGMM estimator**

|  |  | **MGMM/MSMM RR (95%CI) for** | | |
| --- | --- | --- | --- | --- |
|  | **Number non-asthmatic / asthmatic / non-atopic asthmatic / atopic asthmatic** | **Current asthma** | **Non-atopic asthma^a^** | **Atopic asthma^a^** |
|  |  | **at 7½ years^a^** | **at 7½ years^a^** | **at 7½ years^a^** |
| BMI (kg/m^2^) at 7 yrs | 4241/594/249/252 | 2.34 (1.30, 4.24), p=0.005 | 1.97 (1.11, 3.52), p=0.02^‡^ | 1.37 (0.79, 2.36), p=0.26 |
| Males | 2107/352/133/158 | 1.52 (0.86, 2.69), p=0.15 | 1.62 (0.74, 3.56), p=0.23 | 1.21 (0.71, 2.07), p=0.49 |
| Females | 2134/242/116/94 | 1.51 (0.88, 2.58), p=0.14^‡^ | 1.32 (0.79, 2.21), p=0.29^‡^ | 2.03 (1.03, 4.02), p=0.04 |
| Fat mass (kg/2) at 9 yrs* | 3586/500/209/216 | 1.75 (1.19, 2.55), p=0.004^†^ | 1.51 (0.92, 2.48), p=0.10^‡^ | 1.30 (0.73, 2.31), p=0.38 |
| Lean mass (kg) at 9 yrs* | 3586/500/209/216 | 1.55 (0.99, 2.44), p=0.06 | 2.14 (1.38, 3.32), p=0.001 | 1.19 (0.82, 1.73), p=0.35 |
|  |  | **at 9 years^b^** | **at 9 years^b^** | **at 9 years^b^** |
| BMI (kg/m2) at 7 yrs | 3597/701/286/300 | 1.58 (0.93, 2.68), p=0.09^‡^ | 2.89 (1.53, 5.46), p=0.001 | 1.29 (0.77, 2.15), p=0.33^‡^ |
| Males | 1775/414/147/193 | 1.18 (0.69, 2.03), p=0.55^‡^ | 0.77 (0.40, 1.48), p=0.44^‡^ | 0.77 (0.45, 1.30), p=0.33^‡^ |
| Females | 1822/287/139/107 | 1.38 (0.82, 2.31), p=0.23 | 1.50 (0.87, 2.59), p=0.14 | 1.64 (0.79, 3.38), p=0.18 |
| Fat mass (kg/2) at 9 yrs* | 3133/602/244/267 | 1.50 (1.00, 2.26), p=0.05^†^ | 1.29 (0.85, 1.94), p=0.23^†^ | 1.63 (0.92, 2.90), p=0.09^‡^ |
| Lean mass (kg) at 9 yrs* | 3133/602/244/267 | 1.73 (1.11, 2.68), p=0.014 | 1.89 (1.02, 3.48), p=0.04 | 1.54 (0.95, 2.50), p=0.08^‡^ |

* Based on residuals from regression models of mass measure (fat/lean mass) on height, height squared, and gender. Fat mass was divided by 2 so that its standard deviation was similar to that of BMI and lean mass.

^a^ Controls were children with no current asthma at 7½ years

^b^ Controls were children with no current asthma at 9 years

^‡^ No convergence achieved (no red line crossing zero, although in most cases the estimating equation was closest to 0 at RR= MGMM/MSMM RR)

^†^ Double solutions (RR=1.61, 2.55 for current asthma at 7½ years, RR=1.44, 2.76 for current asthma at 9 years, RR=1.23, 4.27 for non-atopic asthma at 9 years)
